# Chasing perception in domestic cats and dogs

**DOI:** 10.1007/s10071-022-01643-3

**Published:** 2022-07-03

**Authors:** Judit Abdai, Stefania Uccheddu, Márta Gácsi, Ádám Miklósi

**Affiliations:** 1 MTA-ELTE Comparative Ethology Research Group, Budapest, Hungary; 2 Department of Ethology, Eötvös Loránd University, Budapest, Hungary

**Keywords:** Cat, Dog, Animacy perception, Chasing, Comparative perception

## Abstract

Chasing motion is often used to study the perception of inanimate objects as animate. When chasing interaction and independent motions between two agents are displayed simultaneously on a screen, we expect observers to quickly perceive and recognise the chasing pattern (because of its familiarity) and turn their attention to the independent motion (novelty effect). In case of isosceles triangles as moving figures, dogs and humans both display this behaviour, but dogs initially preferred to look at the chasing pattern whereas humans started to increase their gaze towards the independent motion earlier. Here, we compared whether family cats perceive moving inanimate objects as animate and whether their looking behaviour is similar to that of small family dogs. We displayed a chasing and independent motion side by side on a screen in two consecutive trials and assessed subjects’ looking behaviour towards the motions. Similarly to previous studies, we found that dogs eventually looked longer at the independent motion, but cats preferred to look at the independent motion at the beginning of the video display and only later shifted their attention to the chasing motion. No difference was found in the frequency of gaze alternation of the two species. Thus, although cats discriminate between the chasing and independent motions, it is not clear whether this discrimination is controlled by animate motion cues. The difference may originate from their ecological situation and/or may be explained by specific perceptual mechanisms.

**Supplementary Information:**

The online version contains supplementary material available at 10.1007/s10071-022-01643-3.

## Introduction

Certain motion patterns can trigger the impression of animacy even in the absence of an animal-like body (animacy perception) (e.g. Scholl and Tremoulet 2000; Tremoulet and Feldman 2000). Perception can be evoked by even a single moving geometric figure that changes its speed and direction simultaneously without visible external cause, which has been observed in newborn human infants (Di Giorgio et al. 2017, 2021) and newly hatched chicks (*Gallus gallus*) (Mascalzoni et al. 2010; Rosa-Salva et al. 2016). Based on the similarities between chicks and humans, it has been suggested that this skill relies on an evolutionary ancient perceptual mechanism at least among vertebrates (Mascalzoni et al. 2010; Rosa-Salva et al. 2016). Rapid animate–inanimate distinction is advantageous in a wide range of contexts, including feeding, social interactions and avoidance of danger. Considering the importance of quick perception of animate entities, it is expected that the motion itself can trigger such perception irrespective of the object/agent displaying the motion or the context in which the movement is presented.

Detection of animate motion is important for both dogs and cats when hunting, and the visual system of both species is adapted to predatory lifestyle (e.g. Bradshaw et al. 2012; Miklósi 2015; Shajid Pyari et al. 2021). However, domestic cats (*Felis silvestris catus*) and their close relatives are mostly solitary hunters, whereas dogs and wolves are pack hunters (but see Marshall-Pescini et al. 2017, describing differences of the feeding ecology of dogs and wolves). This may lead to differences in visual strategies, as dogs (wolves) need to monitor the motion of both the prey and group mates during hunting, whereas cats focus (almost) solely on the pursued prey (for differences in their searching strategies, see Dumas and Dorais Pagé 2006). Thus, dogs (wolves) are used to seeing the motion of a chaser and a chasee in interaction from a third-party point of view, whereas although cats participate in chasing, as a solitary hunter they are probably less exposed to a third-party view of a chasing event.

Chasing is often used as an animate motion pattern because several characteristics of this motion can elicit the perception of inanimate objects as animate (although perception of agency cannot be excluded (e.g. Abdai et al. 2017a, 2021; Gao and Scholl 2011). In previous studies, we displayed simultaneous chasing and independent motions demonstrated by geometric figures to dogs and adult humans (Abdai et al. 2017b, 2021). In both studies, subjects eventually gazed longer at the independently moving figures, probably following the rapid perception of the chasing motion. However, we found differences in the initial behaviour of the two species when using dots as moving figures (Abdai et al. 2017b) versus isosceles triangles, the main axis of which was aligned with the direction of motion (Abdai et al. 2021). In case of dots, dogs and adult humans did not show initial preference to either of the patterns, but in case of the triangles/heading alignment, humans displayed increasing visual attention towards the independent motion from the beginning of the test, whereas dogs initially preferred to look at the chasing pattern. Thus, although both species displayed looking preference towards the independent motion eventually, they reacted differently to the addition of the heading alignment. It was proposed that ecological background and differences in the general visual strategies of dogs and humans could be responsible for such behavioural differences (Abdai et al. 2021). Dogs as a predator species may be highly sensitive to chasing, influencing their initial looking preference towards the chasing motion. However, differences in the behaviour of dogs and humans could also be the result of basic differences in the visual system of the two species. This might influence the frequency of gaze switching between patterns leading to general differences in the behaviour of the two species (see also Park et al. 2020).

Studies on motion detection in cats show that they are able to discriminate between geometric shapes with varying number of sides (polygons) from a circle, displayed on a screen (Berkley and Sprague 1979), and the shape of the moving figure (dot or line) does not influence perception (Berkley et al. 1978; Pasternak and Merigan 1980). Thus, we suggest that geometric figures used in our previous studies (Abdai et al. 2017b, 2021) could be used in studying chasing perception in cats as well. A study, based on the data of two cats, indicated that they perceive biological motion from point-light display (Blake 1993); however, we have no further information regarding their perception of social stimuli (e.g. perception of animacy or agency).

In our previous study, we suggested that the difference found between dogs and humans might be the result of differences in their visual system and thus their visual strategy (differences in the visual exploration of the environment) (Abdai et al. 2021). Based on their visual system, we would not expect marked differences between dogs and cats in the perception of animacy. Cats have an area centralis instead of a fovea, similarly to dogs (Bradshaw 2016). This allows better peripheral vision despite their overall visual field is ca. 200° which is more similar to that of humans (ca. 180°) and brachycephalic dogs.

Here we tested whether cats, similarly to dogs, perceive moving inanimate objects as animate. We displayed the same simultaneous chasing and independent motions of isosceles triangles on two sides of a screen, to pet cats and dogs. We used the same set of stimuli as in our previous study, where we found differences in the looking behaviour of dogs and humans (Abdai et al. 2021). We hypothesised that cats, as mammals, also perceive inanimate objects as animate based simply on their motion. We expected that cats behave similarly to dogs, that is, initially prefer to look at the chasing pattern and only shift their attention to the independent motion later. Alternatively, cats might show increased visual preference for the chasing pattern throughout the test because, as a predator species, chasing is of great importance for cats. Due to similarities in their visual system, we expected a similar frequency of gaze switch between the patterns in cats and dogs.

## Methods

### Ethical statement

Ethical approval was obtained from the National Animal Experimentation Ethics Committee for both cats and dogs (PE/EA/1550-5/2019). All methods were carried out in accordance with relevant guidelines and regulations, and the experiment was performed in accordance with the EU Directive 2010/63/EU. Owners provided a written informed consent to voluntarily permit their cats and dogs to participate in the study.

### Subjects

#### Cats

All tested cats were habituated to the test room in an earlier, separate occasion; the criteria for being ready for the testing was that the cat should accept food from an unfamiliar female experimenter or play with her. The habituation time varied between subjects depending on the cat’s behaviour. It could last less than 10 min with a subject that was keen to explore the environment and interact with (play with and/or accept food from) both the owner and experimenter, or until the cat interacted with both persons (up to 29 min). We tested 42 cats, out of which 12 had to be excluded: 1 cat did not leave the box; the owner of 1 cat was not able to hold the cat in her lap; 5 cats tried to escape from the owner’s hands throughout Trial 2; 2 cats looked at the stimuli for less than 1 s in either of the trials; 1 cat looked at the screen for less than 3 s and in the remaining time the owner moved the cat, thus the gaze of the cat could not be coded; and the gazing of 2 cats could not be coded due to the quality of the video recording. Thus, 30 cats were included in the statistical analyses (1 Maine Coon, 1 Siamese and 28 mixed breed cats; 12 females; mean ± SD age 2.4 ± 2.2 years) (see Online Resource 1).

#### Dogs

We tested 61 dogs, the adult size of which was less than 40 cm high withers. We excluded 24 dogs because they looked at the screen for less than 1 s in either of the trials: 6 dogs because the owner influenced the dog’s behaviour (e.g. forced the dog to look at the screen and hold its head in one position, or pointed at the screen); 2 dogs due to technical issues; and 1 dog because its gaze could not be coded based on the video recording. Thus, 28 dogs were included in the statistical analyses (1 Bolognese, 1 Chinese crested, 1 dachshund, 2 French bulldogs, 1 Havanese, 1 Maltese, 2 miniature dachshunds, 1 miniature pinscher, 1 miniature poodle, 3 miniature Schnauzers, 1 Papillon, 3 Yorkshire terriers, 2 mixed breed dogs and 8 mongrels; 17 females; mean ± SD age 4.7 ± 3.5 years) (see Online Resource 1).

### Apparatus

**Fig. 1:**
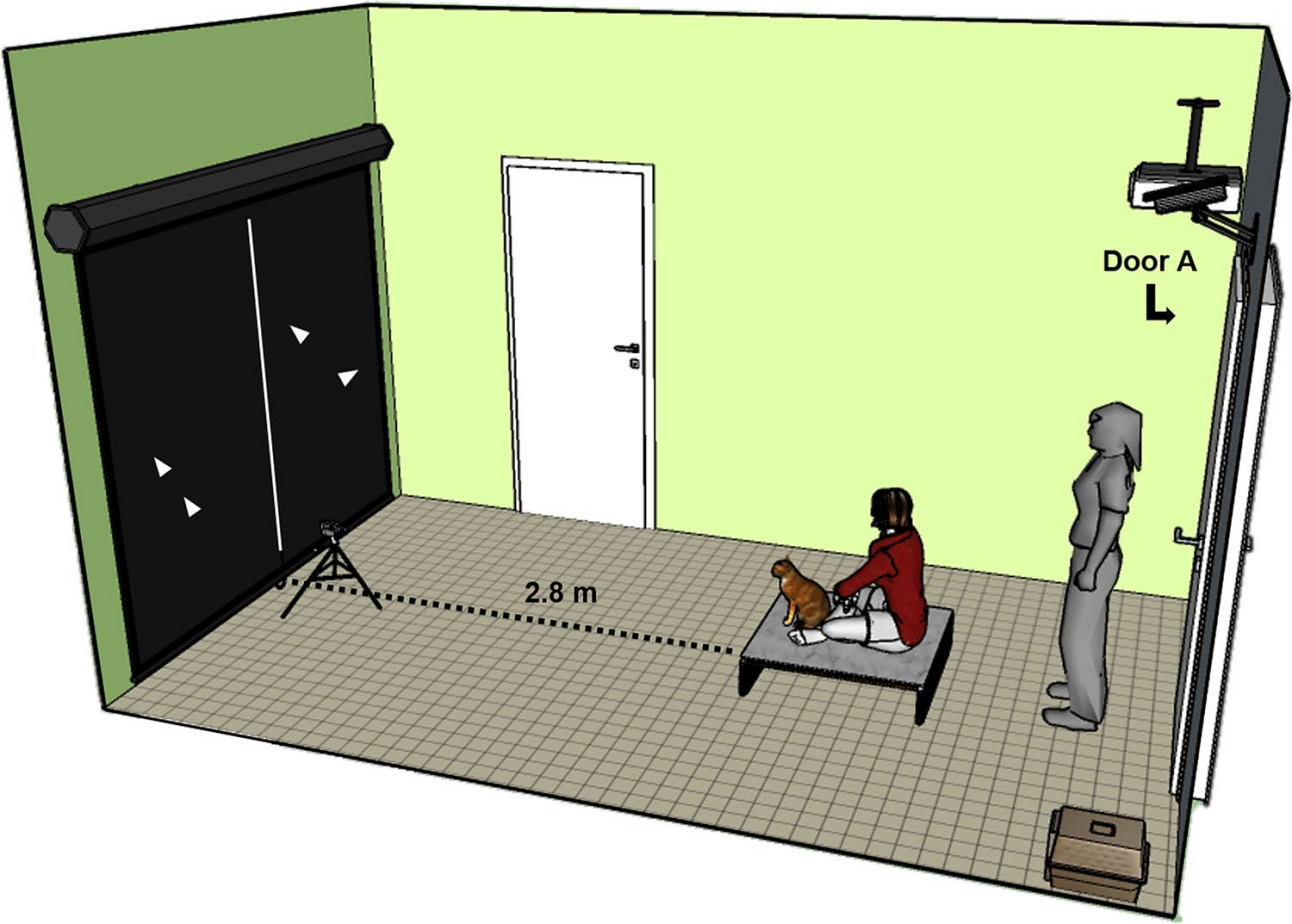
Experimental setup of the room. In case of dogs, the box was not present in the room (dogs were led on leash by their owner), and the experimenter was not present in the room in most cases

Subjects were tested at the Department of Ethology, Eötvös Loránd University, in a 5.2 m × 3 m testing room. Tests were recorded with two synchronised cameras. One camera was mounted on the ceiling behind the subjects, focusing on the video displays. The other camera was a 25 frame per second zero lux camera (Sony FDR-AX53) mounted on a compact tripod placed before the screen, equidistant from its sides, focusing on the face of subjects. Infrared LEDs placed next to the camera were directed towards the subjects to improve eye visibility. The projector was mounted on the ceiling behind subjects. Audio was displayed by two speakers centred behind the screen to avoid possible asymmetric cues. Videos were displayed on a screen (2 m × 2.1 m) placed 2.8 m in front of the subject (Fig. 1).

### Procedure

The owner entered the room (door A) along with Experimenter 1 (E1), bringing the cat inside their box and dogs were led in the room on leash. The owner placed the box of the cat on the ground, in the corner next to the door and opened it. Dogs were released from the leash. Cats and dogs could explore the room. After the exploration, the owner sat on a wooden platform covered with artificial grass (H × W × L: 25 cm × 80 cm × 80 cm) and held the subject in his/her lap facing the screen (Fig. 1). E1 adjusted the focus of the zero lux camera to capture the subjects’ face and turned off the lights next to door A. In the case of cats and some dogs (*N* = 9), E1 stayed there motionless during the display of the video and Experimenter 2 (E2) started the display from the adjacent room. For most dogs (*N* = 19), there was only one experimenter because the other experimenter could not participate in the testing anymore. Thus, after turning the lights off, E1 left the room and started the video from the adjacent room.

We used the same set of videos as in Abdai et al. (2021) that consisted of the following: (1) 2.32 s-long audiovisual attention grabber directing subjects’ attention to the centre of the screen, (2) 10 s stimulus (Trial 1), (3) plain black screen for 3 s, (4) 2.32 s audiovisual attention grabber, and (5) 10 s stimulus (Trial 2) (see Online Resource 4). All subjects saw one unique video. Videos were generated by the ChasingDots program (developed by Bence Ferdinandy; Abdai et al. 2017b). Stimuli were dependent (henceforth ‘chasing’) and independent movement patterns of two white isosceles triangles presented side by side, over a plain black background separated by a white vertical line in the middle of the screen. In the independent patterns, one figure was a chaser and the other a chasee from two different chasing patterns; thus, the motion dynamics of the chasing and independent patterns were the same. The sides of the chasing and independent patterns were counterbalanced between trials and subjects.

### Data analyses

All tests were recorded and subjects’ behaviour was analysed with Solomon Coder 19.08.02 (developed by András Péter: http://solomoncoder.com). Data were analysed by R software version 4.1.1 [R Development Core Team (2021)] in RStudio version 1.4.1717. Backward model selections were carried out using drop1 function; selection was based on the likelihood ratio test (LRT). LRTs of non-significant variables were reported before their exclusion from the models. For significant explanatory variables in the final models, we carried out pairwise comparisons (‘emmeans’ package) and we report contrast estimates (*β* ± SD).

All videos were coded frame by frame (25 frames per second); for each frame, gaze direction (independent, dependent, away) was determined. Looking duration at the patterns was coded based on eye movements. Inter-coder reliabilities on random subsamples (20% of cats and 20% of dogs) indicated acceptable reliability (mean ± *SD* Cohen’s kappa: cats, 0.767 ± *0.167;* dogs, 0.742 ± *0.053*).

Looking duration of subjects was analysed using linear mixed model (LMM; ‘lme4’ package). Residuals of the model were normally distributed after Tukey’s ladder of power transformation (‘rcompanion’ package; lambda 0.65) (Shapiro–Wilk test: *W* = 0.993, *p* = 0.351). We estimated the fixed effects of motion pattern (chasing vs independent), trial (trial 1 vs 2) and species (cat vs dog) (three-way interaction). We also tested whether the pattern subjects looked at first in the specific trial or whether the side on which the chasing pattern was displayed had an effect on their looking behaviour. The subjects’ IDs were included as a random intercept to control for within-subject comparison across trials. We also included Trial and Pattern as random slopes to account for the non-independence of the data.

For each trial, we created looking-time curves of cats and dogs to investigate overall within-trial dynamics of gazing at the screen when the stimuli were displayed (Python 3.7.6 in Jupyter Notebook 6.0.3). A single point of a curve represents the proportions of time spent looking at the chasing and independent patterns for every three consecutive frames in the specific trial, separately for cats and dogs. Considering that at the onset of the trial subjects did not look at the stimuli, we only included data points after the proportion values reached 80% of the average proportion of looking time at the stimuli during the specific trial. Linear regression was applied to the data to capture overall trends and estimate slopes (*β* ± SE).

We counted the frequency of gaze shifts between patterns, irrespective of delays in between. Based on the AIC values (model comparison with ANOVA), Poisson distribution fit the data best (AIC = 476.86; model with the lowest AIC value was kept and a model was considered better whenever ΔAIC was ≥ 2). We carried out generalised linear mixed model (GLMM; ‘lme4’ package) to analyse the data. We estimated the fixed effects of Trial (trial 1 vs 2) and Species (cat vs dog) (two-way interaction), and included the ID of subjects as a random effect.

## Results

**Fig. 2:**
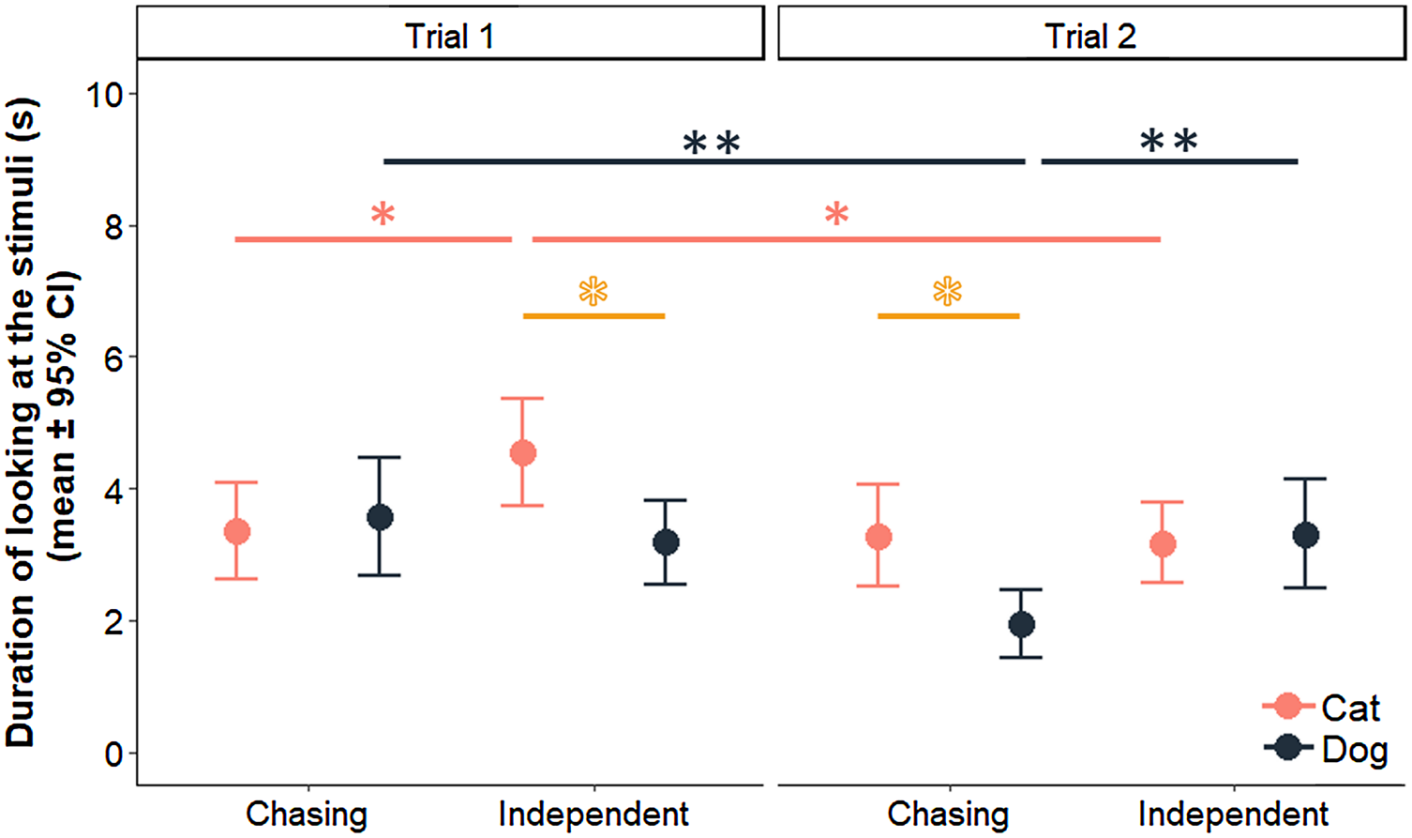
Duration of looking at the chasing and independent patterns in Trials 1 and 2 by cats (pink) and dogs (blue). Pink (light) lines indicate within-subject differences in cats, blue (dark) lines indicate within-subject differences in dogs, and yellow (medium grey) lines with empty asterisks indicate between-species differences; *p < 0.05, **p < 0.01 (colour figure online)

We found a significant three-way interaction between Species, Trial and Pattern for the looking duration of subjects (LMM: Species × Trial × Pattern, ${\chi }_{1}^{2}$ = 7.866, *p* = 0.005) (result of pairwise comparison is presented in Table 1 and on Fig. 2). In comparison with dogs, cats looked longer at the independent motion in Trial 1, and at the chasing pattern in Trial 2, but no difference was found in the case of the chasing pattern in Trial 1 or the independent motion in Trial 2. Cats looked at the independent motion significantly longer than at the chasing pattern in Trial 1, and they looked at this motion longer in Trial 1 than in Trial 2. However, there was no difference in the looking duration of cats towards the two patterns in Trial 2, and we did not find difference in their looking duration towards the chasing pattern between trials.

**Table 1:** Effect of species, trial and pattern interaction on subjects’ looking duration (LMM based on LRT; pairwise comparison using ‘emmeans’ package); we provide contrast estimates (*β* ± SE). Highlights indicate significant variables

Species	Trial	Pattern	*β* ± SE	*p* value
Between patterns				
Cat	1	Chasing vs independent	− 0.520 ± 0.223	0.022
	2		0.002 ± 0.223	0.994
Dog	1		0.136 ± 0.231	0.558
	2		− 0.607 ± 0.231	0.010
Between species				
Cat vs Dog	1	Chasing	− 0.091 ± 0.227	0.689
		Independent	0.564 ± 0.227	0.015
	2	Chasing	0.595 ± 0.227	0.010
		Independent	− 0.014 ± 0.227	0.951
Between trials				
Cat	1 vs 2	Chasing	0.039 ± 0.223	0.863
Dog			0.725 ± 0.231	0.002
Cat		Independent	0.560 ± 0.223	0.013
Dog			− 0.019 ± 0.231	0.936

Dogs did not display preference to either of the patterns in Trial 1, but looked longer at the independently moving figures in Trial 2. Dogs looked at the chasing pattern longer in Trial 1 than in Trial 2, but no difference was found in their looking duration towards the independent motion across trials.

The pattern subjects looked at first, and the side of the chasing pattern had no effect on the looking duration (LMM: first look, ${\chi }_{1}^{2}$ = 0.662, *p* = 0.416; side, ${\chi }_{1}^{2}$ = 0.681, *p* = 0.409).

### Within-trial dynamics of looking at the stimuli

**Fig. 3:**
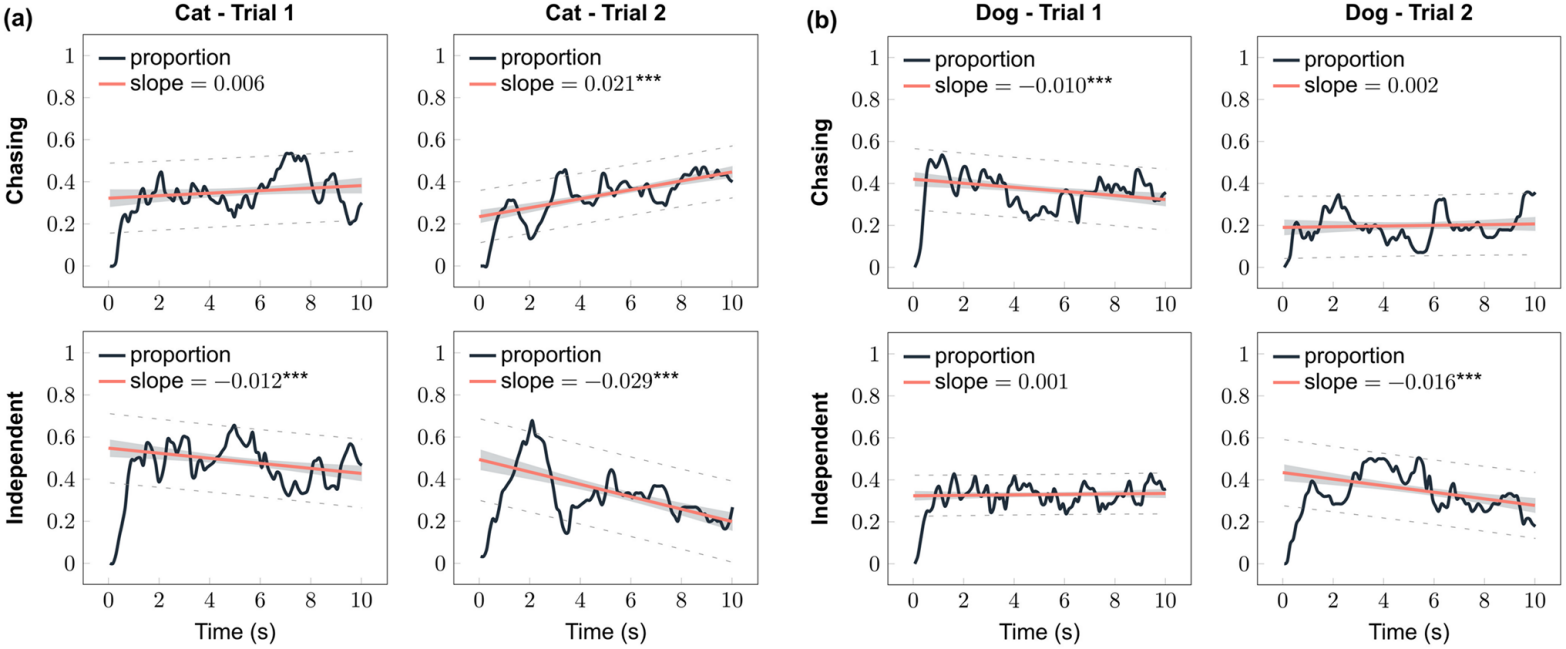
Proportions of looking at the chasing and the independent patterns in **a** cats and **b** dogs. Grey area indicates 95% confidence interval, and dashed lines indicate the 95% prediction interval. Regression lines were fitted using data after the proportion values reached the 80% threshold computed from the average proportion of looking time at stimuli during the trial for the subject. *** *p* < 0.001 (colour figure online)

Mean proportion of cats’ gaze towards the independent pattern significantly decreased in Trial 1, but showed a marginal increase towards the chasing pattern (cat, Trial 1: chasing, *ß* ± SE = 0.006 ± 0.004, *p* = 0.094; independent, *ß* ± SE = − 0.012 ± 0.003, *p* < 0.001) (Fig. 3a). Their gaze at the independent pattern continued to decrease, but they increased their look significantly towards the chasing pattern during Trial 2 (cat, Trial 2: chasing, *ß* ± SE = 0.021 ± 0.003, *p* < 0.001; independent, *ß* ± SE = − 0.029 ± 0.004, *p* < 0.001) (Fig. 3a). This suggests that lack of overall difference in their looking duration towards the two patterns in Trial 2 (see above) is the result of their gaze preference changing to opposite throughout the trial.

In Trial 1, dogs decreased their gaze at the chasing motion, but no change was found in their visual attention to the independent motion (dog, Trial 1: chasing, *ß* ± SE = − 0.010 ± 0.003, *p* = 0.001; independent, *ß* ± SE = 0.001 ± 0.002, *p* = 0.571) (Fig. 3b). However, this changed for Trial 2, that is, dogs’ gaze at the chasing motion did not change while continuously decreased their look towards the independent motion (dog, Trial 2: chasing, *ß* ± SE = 0.002 ± 0.003, *p* = 0.601; independent, *ß* ± SE = − 0.016 ± 0.003, *p* < 0.001) (Fig. 3b).

### Gaze alternation between patterns

A two-way Species by Trial interaction had no effect on the frequency of gaze alternation between patterns (GLMM with Poisson distribution: Species × Trial, ${\chi }_{1}^{2}$ = 0.007, *p* = 0.935; mean ± SD: Trial 1, cats, 4.3 ± 2.6; dogs 4.1 ± 2.0; Trial 2, cats 2.7 ± 1.5; dogs, 2.5 ± 1.6). Cats and dogs shifted their gaze similarly between the two motion patterns (Species, ${\chi }_{1}^{2}$ = 0.202, *p* = 0.653). We found a significant difference between trials (Trial, ${\chi }_{1}^{2}$ = 21.570, *p* < 0.001), indicating that both species exhibited more frequent gaze alternation in Trial 1 than in Trial 2 (Trial 1 vs 2, *ß* ± SE = 0.473 ± 0.103, *p* < 0.001).

## Discussion

Cats discriminated between dependent and independent motions of geometric figures; however, they displayed different behaviour from predicted. We hypothesised that in the case of perceiving the dependent motion as animate (or as chasing), subjects would either show no initial interest or prefer to look at the chasing pattern, whereas they would gradually increase their look towards the independent pattern (see Abdai et al. 2017b, 2021; Rochat et al. 1997). Dogs initially displayed no looking preference towards either of the motion patterns, but later decreased their look towards the chasing motion while keeping their visual attention on the independently moving figures. Cats’ looking pattern was the opposite of what we had expected. They displayed an initial preference towards the independent motion and then gradually decreased their gaze towards this motion, whereas they displayed increasing visual attention towards the chasing pattern. Cats and dogs shifted their gaze between patterns with similar frequency; thus, this aspect of the visual strategy could not drive the looking preference here (cf. our previous study on dog–human comparison; Abdai et al. 2021). Although cats displayed different looking behaviour towards the patterns, we can only speculate about what controlled their behaviour. We included several different dog breeds (including mixed breeds and mongrels) to avoid the potential influence of selection for specific behaviour traits, or the influence of head morphology, so our results have general implication in dogs.

Due to the matched dynamics of the moving figures on both sides (chaser and chasee), the only difference between the patterns is in the dependency of their motion in the chasing version; thus, one might argue that our results still support the perception of animacy in cats. However, considering that in the present study cats displayed higher visual attention towards the independently moving figures from the first seconds of the test, we cannot argue that cats’ look was driven by the rapid perception of the chasing motion.

Dogs (wolves) are hunting in groups; thus for them, not only the motion of the prey, but also the motion of the pack members is important to follow (Dumas and Dorais Pagé 2006). Although chasing motion is ecologically relevant for cats, as solitary hunters (Cecchetti et al. 2021) they may be less exposed to observing a chasing event as a third party, or looking at the motion of the chaser and chasee might indicate an “occupied” prey for them. It should also be mentioned that the motion of both the chaser and chasee involved characteristics attributed to animacy (e.g. sudden changes in speed and direction). Thus it is possible that instead of a chasing and independent motion patterns, cats perceived the four figures on the screen as four different animate objects (or three considering the small interobject spacing in the chasing interaction), and their visual preference was split among these potential preys. Dumas and Dorais Pagé (2006) suggested that due to cats pursuing only one prey at a time, a second target can distract them. On our display, the independent motion might have presented two potential preys that was interesting initially, but over time cats gazed more at the chasing where the two figures had similar trajectories and thus caused less distraction.

Differences in the hunting style of dogs and cats may explain why the two species showed different behaviour in the present study (see also Lõoke et al. (2020) on how hunting style of the two species may cause differences in their motion perception). However, in a recent study, Abdai and Miklósi (2022) found that hunting dogs selected for track and chase prey versus hunting dogs selected to mark and remember downed game distributed their visual attention similarly between the chasing and independent motion patterns.

The visual field of cats is ca. 200°, while in dogs it varies between 200° and 250° depending on the skull length (Bradshaw 2016; Miller and Murphy 1995). In our study, we tested dogs with different skull lengths to control for the potential effect of the head shape; however, head shape might influence dogs’ behaviour (cf. cats whose visual field is similar to that of brachycephalic dogs). Although we have no information about its effect in social perception, short-headed dogs are more attentive to faces (Bognár et al. 2018) and perform better when using human visual cues (Gácsi et al. 2009). More studies are needed to explore whether selection for other behaviour traits and differences in the distribution of ganglion cells influence the perception of animate/chasing motion.

Thus, based on the present findings, domestic cats discriminate between a chasing and independent motion pattern, but we cannot argue that cats perceived the chasing pattern in a way as dogs and humans do. We cannot exclude that in cats either the (1) motion parameters applied here or (2) applying video projection as research method might be inappropriate to study the phenomenon. First, we applied the same stimuli that we used to study dogs and humans (Abdai et al. 2021), but these specific parameters (e.g. speed or minimum turning angle) could fail to elicit animacy perception in cats, or other aspects of the display (e.g. spatial distance between moving figures) masked this perception. Considering how attentive cats are to moving stimuli in their daily lives (e.g. laser pointer), it is possible that they are more reactive to motion that overshadows animacy perception as defined by our concept (see above). Although there was a difference in the mean age of our dogs and cats that may influence their previous experiences, we assume that all subjects had exposure to chasing motion before. We tested whether companion animals and owners frequently play with both dogs and cats in a way that includes chasing (e.g. chasing a ball or a moving toy) from puppyhood/kittenhood. Thus, difference in exposure to chasing is unlikely to cause a significant influence.

Second, video displays are widely used in non-human species to present specific stimuli (for a review, see e.g. D’Eath 1998); nonetheless, it may not be the best approach in all cases due to the differences in the vision of species and the artificial context (see D’Eath 1998; Fleishman and Endler 2000). Looking duration of cats and dogs towards the screen decreased throughout both trials (see also Abdai et al. 2017b, 2021 for dogs), which is likely due to reduced interest in the stimuli or the artefact of the method used. Further, here cats and dogs were not trained to stay in one position or to look at the screen. Several cats had to be excluded because they tried to escape from the owner’s hand during the test, and in one case the owner could not hold the cat long enough to start the video projection (see Online Resource 1). This suggests that displaying stimuli on a screen while being held in one position by a human might influence cats’ viewing behaviour here (although unlikely to have an effect on the looking preference itself). Thus, it may be advantageous to demonstrate the events in a more realistic context, while cats can move freely during observation (or at least not held by a human) in future studies (see e.g. Abdai et al. 2017a). Utilisation of artificial agents (robots) as moving objects would allow testing not only cats’ looking preferences, but also facilitate the measurement of other behaviours displayed directly towards the animate/inanimate objects. Live demonstration of the motion may also be more interesting for cats (or non-human species in general). The use of robots also enables researchers to control for specific motion cues to explore their effect on the perception of the moving object as animate (Abdai et al. 2017a, 2018).

Our study revealed interesting differences between dogs and cats regarding their perception of a chasing motion performed by inanimate virtual objects. Our results did not confirm that cats react to similar stimuli as dogs and humans regarding animacy perception. The difference may originate from their ecological situation and/or may be explained by specific perceptual mechanisms.

## Supplementary Information

Below is the link to the electronic supplementary material.

Supplementary file1 (XLSX 34 KB)

Supplementary file2 (TXT 3137 KB)

Supplementary file3 (TXT 1602 KB)

Supplementary file4 (MP4 58816 KB)

## Data Availability

All data generated or analysed during this study are included in this published article and its supplementary information files.
